# BrainFit: improving executive and subjective cognitive functioning in late-life mood disorders - a double-blind randomized active-controlled study evaluating the effect of online cognitive training

**DOI:** 10.3389/fpsyt.2024.1509821

**Published:** 2025-01-02

**Authors:** Mardien L. Oudega, Margot J. Wagenmakers, Tanya Palsma, Adriaan W. Hoogendoorn, Chris Vriend, Odile A. van den Heuvel, Sigfried Schouws, Annemiek Dols

**Affiliations:** ^1^ GGZ inGeest Specialized Mental Health Care, Amsterdam, Netherlands; ^2^ Department of Psychiatry, Amsterdam UMC location Vrije Universiteit Amsterdam, Amsterdam, Netherlands; ^3^ Amsterdam Neuroscience, Mood, Anxiety, Psychosis, Stress and Sleep Program, Amsterdam, Netherlands; ^4^ Amsterdam Public Health (Research Institute), Amsterdam UMC, Vrije Universiteit, Amsterdam, Netherlands; ^5^ Department of Psychiatry, Amsterdam UMC, Vrije Universiteit Amsterdam, Amsterdam, Netherlands; ^6^ Department of Anatomy & Neuroscience, Amsterdam UMC, Vrije Universiteit Amsterdam, Amsterdam, Netherlands; ^7^ Compulsivity Impulsivity Attention Program, Neurodegeneration Program, Amsterdam Neuroscience, Amsterdam, Netherlands; ^8^ Department of Psychiatry, UMC Utrecht Brain Center, University Medical Center Utrecht, Utrecht, Netherlands

**Keywords:** cognitive training, unipolar depressive disorder, bipolar disorder, executive functioning, cognitive impairment, cognition, older adults, late-life mood disorders

## Abstract

**Introduction:**

Unipolar and bipolar mood disorders in older adults are accompanied by cognitive impairment, including executive dysfunction, with a severe impact on daily life. Up and till now, strategies to improve cognitive functioning in late-life mood disorders (LLMD) are sparse. Therefore, we aimed to assess the efficacy of adaptive, computerized cognitive training (CT) on executive and subjective cognitive functioning in LLMD.

**Methods:**

In this double-blind, randomized controlled study we enrolled patients over the age of 50 with partly remitted LLMD. Over 8 weeks, patients participated in 24 45-minute sessions of computerized multi-domain training (CT) or an active control condition (ACC) (nonspecific cognitive activity). The primary outcome was executive functioning based on the interference score on the STROOP task (not incorporated in the training). Secondary outcomes were subjective cognitive functioning, depressive symptoms and quality of life. Outcomes were assessed before and after training (T1) and at a 3-month follow-up (T2) and analyzed with linear mixed-model analyses.

**Results:**

Thirty-eight patients were included in the study, 22 in the experimental CT and 16 in the ACC. Mean age was 67.3 years and 52.6% was female. Linear mixed-model analyses showed small within-group effect sizes, corresponding to no statistically significant improvement of executive functioning or depression severity in either group. In both groups we did observe an improvement on subjective cognitive functioning over time. From T0 to T1 the mean score of the Cognitive Functioning Questionnaire (CFQ) of the CT group decreased from 52.7 to 46.8 points (p=0.003) and the mean CFQ score of the ACC group decreased from 52.7 to 45.7 points (p<0.001). This effect remained in both groups at follow-up (T2); respectively p=0.002 and p<0.001.The patients in the AAC also showed an improvement of quality of life directly after the training (T1); i.e. the mean quality of life scores improved from 53 to 57 points (p=0.011), but this effect did not remain at follow-up.

**Conclusions:**

This study shows no beneficial effect of an 8-week computerized CT on the primary outcome, i.e, executive functioning. Subjective cognitive functioning did improve in both groups, indicating that frequent cognitive training is advantageous. Future studies with more intensive training could be designed to explore this result further.

**Clinical trial registration:**

clinicaltrials.gov, identifier NCT04006756.

## Introduction

Late life mood disorders (LLMD) concern patients with unipolar depression and bipolar disorder, aged 50 years and over. Despite the fact that evidence-based pharmacological and psychotherapeutic interventions have proven therapeutic effectiveness, many patients with LLMD experience relapse or partial remission (1, 2). One of the reasons for unfavorable treatment outcomes is that LLMD are often accompanied with neurocognitive impairments in the areas of executive functioning, attention, memory and speed of information processing (3–5). during an episode and after remission (6, 7). Cognitive impairment in LLMD is associated with worse social functioning, distress to patients and caregivers, decreased quality of life and an unfavorable prognosis, including nursing home admission (8–10).

A meta-analysis of adult patients with major depressive disorder showed that computerized cognitive training (CT) is associated with improvement in depressive symptoms and everyday functioning, though effects on cognition are inconsistent, with moderate to large effects for attention, working memory and global functioning and no effects for executive functioning and verbal memory (11). Preiss et al. (12) investigated the effect of CT in unipolar and bipolar depression, and they found that participants in the training group reported significantly lower levels of depression, as well as significant improvement in neurocognitive functioning in aspects of executive functioning (set shifting, attention control, and global executive score). However, they did not compare the results with an active control condition, leaving it unclear whether the improvement is due to the online CT or general cognitive engagement. A randomized controlled trial (RCT) by Strawbridge et al. (13) evaluating the effectiveness of cognitive remediation therapy (CRT) compared to treatment as usual (TAU) in 60 patients with bipolar I and II mood disorders confirmed the effectiveness of CRT, with large effect sizes for improvement in working memory (Cohen d = 0.70), general executive functioning (0.93), goal attainment (0.89), and social functioning (0.49) in the intervention group compared to TAU.

In conclusion, studies evaluating the effectiveness of CT are sparse and inconsistent in patients with LLMD, possibly due to differences in CT interventions or study methods and heterogeneity of patients. Interestingly, studies evaluating the effectiveness of CT on executive functioning in patients with other brain diseases, show positive results, e.g., in patients with dementia (14–16), mild cognitive impairment (MCI) (17), schizophrenia (18), and Parkinson’s disease (19). The study in Parkinson’s disease used an online CT tool ‘BrainGymmer’, and showed an improvement of executive functioning after the training. However, this result was not replicated in a large RCT by van Balkom et al. (20), that showed only minor short-term improvements on processing speed.

The primary aim of the current study was to investigate the effects of the online CT on executive functioning in patients with LLMD compared with an active control condition (ACC). The secondary aims were to evaluate the effectiveness of CT on subjective cognitive symptoms, severity of depression and quality of life. We also investigated if benefits from CT persist after a 3-month follow-up. Based on the executive deficits seen in LLMD and the results from previous studies (12, 13, 16–19, 21) we hypothesized that the online ‘BrainGymmer’ intervention would improve the executive functions, as measured with the STROOP task, in the patients in the intervention group (CT) as compared with the ACC group. Furthermore, based on Willis et al. (22) results, we hypothesized that the improvement would still be present 3 months after the CT ended.

## Method

### Study design

BrainFit was a double-blind, randomized controlled trial assessing the effectiveness of an online eight-week computerized CT compared with an active control condition in LLMD. This study was approved by the medical committee of VU University medical center and performed in accordance with the Declaration of Helsinki. All patients provided written informed consent. The trial was prospectively registered at clinicaltrials.gov (NCT04006756). Patients could withdraw at any moment from the study; if this was done before the start of the intervention, a new participant was included as a replacement. If a patient decided to withdraw during the intervention, having followed the intervention for at least 4 weeks, the patient was invited to participate in a final measurement and included in the analyses.

### Study sample

Patients were recruited from September 2019 till November 2021 at the outpatient clinic of a mental health institute in Amsterdam (GGZinGeest). They were at least 50 years of age and suffering subjective cognitive symptoms. We included older aged patients with remitting unipolar depression, in early remission or partly in remission, confirmed by a MINI-interview. Also we included older aged patients with bipolar depression, diagnosed by their treating psychiatrist using DSM-5 (23) and confirmed by a MINI interview. To be eligible for the study, patients needed to comply with the following inclusion criteria: 1) a score higher than 44 on the subjective cognitive functioning questionnaire (CFQ) (24, 25), indicating relevant subjective cognitive symptoms, 2) a recurrent unipolar depression in early or partial remission, confirmed by a MINI-interview or a bipolar disorder diagnosed by DSM-5 (23), 3) home access to a computer, laptop, or tablet with internet access, 4) willing to provide informed consent. Patients were not eligible to participate in the study if any of the following exclusion criteria were present: 1) signs of dementia, indicated by a score lower than 22 on the Montreal Cognitive Assessment (MoCA) (26) or if neuropsychological assessment showed a deficit of more than 1 SD below the norm on two or more cognitive domains, 2) indication of current drug- or alcohol abuse (CAGE AID-interview score >1) (27, 28), 3) unable to undergo neuropsychological examinations, 4) psychotic symptoms, 5) severe suicidal thoughts, 6) a severe personality disorder (as primary diagnosis).

### Procedure

**Figure 1:**
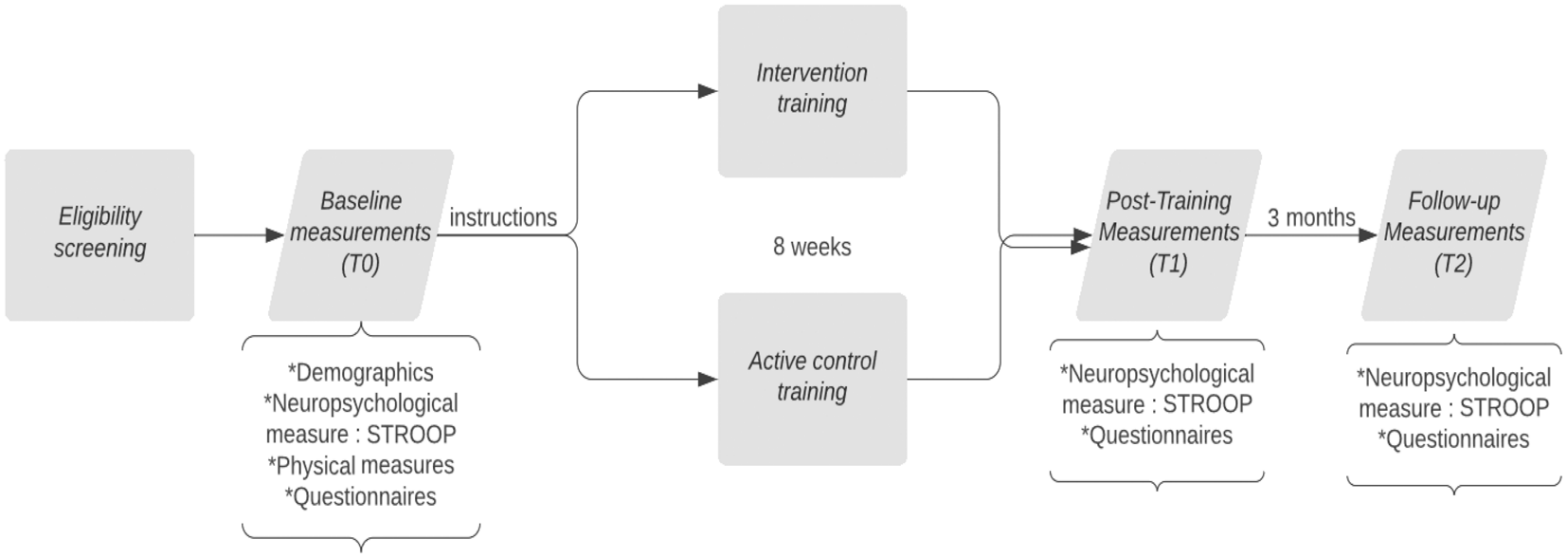
Flowchart of the BrainFit timeline.

After eligibility screening, patients underwent a baseline assessment (T0) that entailed neuropsychological testing, physical measures, and questionnaires, provided by a blinded study member. The following neuropsychological tests were administered: Dutch variant of Rey’s Auditory Verbal Learning Task (RAVLT), Visual association test (VAT), Trailmaking Test (TMT A and B), WAIS-III digits forwards and backwards, Stroop Color Word Test, clock drawing, figure copying (pentagram), Control Oral Word Association Test (COWAT), Animal and Occupation Naming subtest of the Groningen Intelligence Test (GIT), Boston Naming Test and Montreal Cognitive assessment (MOCA). All tests were assessed for their reliability earlier by Egberink et al. (29) and parallel versions were used if available. Thereafter, a non-blinded study member manually allocated the participant to either the CT or the ACC, stratified on education level and polarity of the mood disorder. Next, a non-blinded study member provided the participant with instructions about the log-in, the duration, and the frequency of the training. Patients started with 8 weeks of online CT or ACC, both training at home three times a week. In total, participants trained for twenty-four sessions and each session lasted approximately 45 minutes. After 8 weeks of online training, patients were invited for the post-intervention assessment (T1). Finally, 3 months after finishing the training (T2) patients underwent a neuropsychological assessment again and filled in questionnaires. A flowchart is provided in **Figure 1**. Due to the COVID-19 pandemic, most assessments were performed over the phone or via a video call. This could differ per patient.

### Cognitive training (CT) and active control condition (ACC)

The online cognitive intervention used was a modified version of the BrainGymmer online CT platform (https://www.braingymmer.com/en/, a product by Dezzel Media B.V.). The aim was to train cognitive abilities, such as executive functions (cognitive flexibility, planning inhibition and working memory), attention, and processing speed. The training was available for the patients in the comfort of their own homes.

The CT consisted of 13 games that were sequentially performed by the participants in a pseudorandomized order. The games were substantially different from the neuropsychological tasks done during the neuropsychological assessment, but they do call upon the same cognitive processes. Working memory and mental flexibility were trained and the games were equipped with a ‘dynamic difficulty adjustment,’ thus the difficulty of the games adapted to the participants’ performance. This ensured that the participants were challenged to continuously perform at their maximum ability.

We included the ACC to correct for nonspecific cognitive activity. The participants in the ACC participated in three cognitive engaging games: solitaire, trivia questions, and hangman. The patients sequentially performed these games for 45 minutes each session. These games were hypothesized not to train specific cognitive domains.

### Outcomes

The primary outcome was the efficacy of the online CT (compared to the control condition) on executive functions, as was measured using the STROOP task (30). The STROOP task is a neuropsychological test to assess the ability to inhibit cognitive interference. It measures several aspects of executive functions, including inhibition, attention control, cognitive flexibility and working memory (31). This task consists of three cards, the first has black and white color words and the patient’s goal is to read the words as quickly and accurately as possible. The second has colored blocks, here the patient’s goal is to name the colors of the blocks as quickly and accurately as possible. Finally, the third has colored words written in an incongruent color, the patients are asked to name the color of the words, not read the word itself, as quickly and accurately as possible. Patients acquire an interference score based upon the score on the third card given their score on the second card. This interference score is an indicator for aspects of executive functioning, such as inhibition (31). Unfortunately, error rates were not available, so we could only use the completion time for each condition and based on that calculate the interference score.

The Stroop was used as the primary outcome measure because decrements in information processing speed and executive functioning in older aged patients with mood disorders are associated with poorer IADL performance (32, 33).

To investigate the effect of CT on more daily life aspects, secondary outcome measures included 1) subjective cognitive symptoms, 2) depression severity and 3) quality of life. Subjective cognitive symptoms are measured by the subjective CFQ (25) at baseline, post-training, and follow-up. The CFQ was used as this measure is sensitive to small cognitive errors in daily living such as memory problems (24). The CFQ consists of 25 questions on small everyday mistakes, the items relate to memory and attention. Higher scores on the CFQ indicate more cognitive mistakes. The Manchester Short Assessment of Quality of Life (Mansa) (34) measured quality of life at baseline, post-training, and follow-up. The Mansa consists of 12 subjective questions about daily life, physical health, safety, and financial situations. Higher scores on the Mansa indicate a higher quality of life (35). Finally, depression severity was measured using the Montgomery Åsberg Depression Rating Scale (MADRS) (36), this instrument consists of 10 questions. A higher score indicates more severe depression.

### Statistical analyses

A systematic review of Miskowiak et al. (37) concluded that the primary outcome measure to be used in RCTs evaluating CT in patients with mood disorders should be executive functioning. The sample size calculation of the current study was based on a standardized effect size (SES) = 0.93 of CT on executive functioning as reported in an earlier study of Strawbridge et al. (13). We calculated that the sample size needed to detect the effect (with α = 0.05 and β = 0.80) equaled n = 38, i.e. 19 per group.

Standard statistical measures, mean, and standard deviation were used to present the demographic measures of both groups. General demographic measurements, clinical characteristics, and cognitive measurements of the CT condition and ACC were compared using the appropriate parametric and non-parametric tests, i.e., t-tests, Mann-Whitney U tests, or Chi-square tests. The primary outcome was analyzed using linear mixed models (LMM), with t-transformed STROOP scores (t-transformed meaning: corrected for age, sex, and education) as the outcome variable over time. Utilizing an LMM, it was investigated whether patients participating in the online CT had more improvement in executive functioning compared to the active control condition and whether this improvement was resistant after training by examining the interaction terms between the condition and time. The analyses were corrected for baseline differences of the STROOP scores. We did not control for age, sex, and education level in the LMM, because the t-transformed STROOP scores were already corrected for these variables. Secondary outcomes were also analyzed using LMM, where we investigated whether patients participating in the online CT had improved more on their subjective cognitive functioning, their quality of life, and the severity of their depression as compared to participants of the active control condition. Furthermore, it was investigated whether this improvement remained after training by examining the interaction between time and condition. Assumptions of linearity, multicollinearity, homoskedacity, normality, and independence were checked. Selection of the correct variance-covariance structure (unstructured, compound symmetry, or heterogeneous compound symmetry) was based on likelihood ratio tests and information criteria. Thereafter, the actual LMM with the adequate variance-covariance matrix was estimated using the restricted maximum likelihood method. Pairwise comparisons were analyzed and corrected for multiple comparisons, with Bonferroni (38). Data were analyzed using the Statistical Package of the Social Sciences (SPSS, version 26, SPSS Inc., Chicago, IL). In all analyses, a p < 0.05 was considered statistically significant.

## Results

### Patients

**Figure 2:**
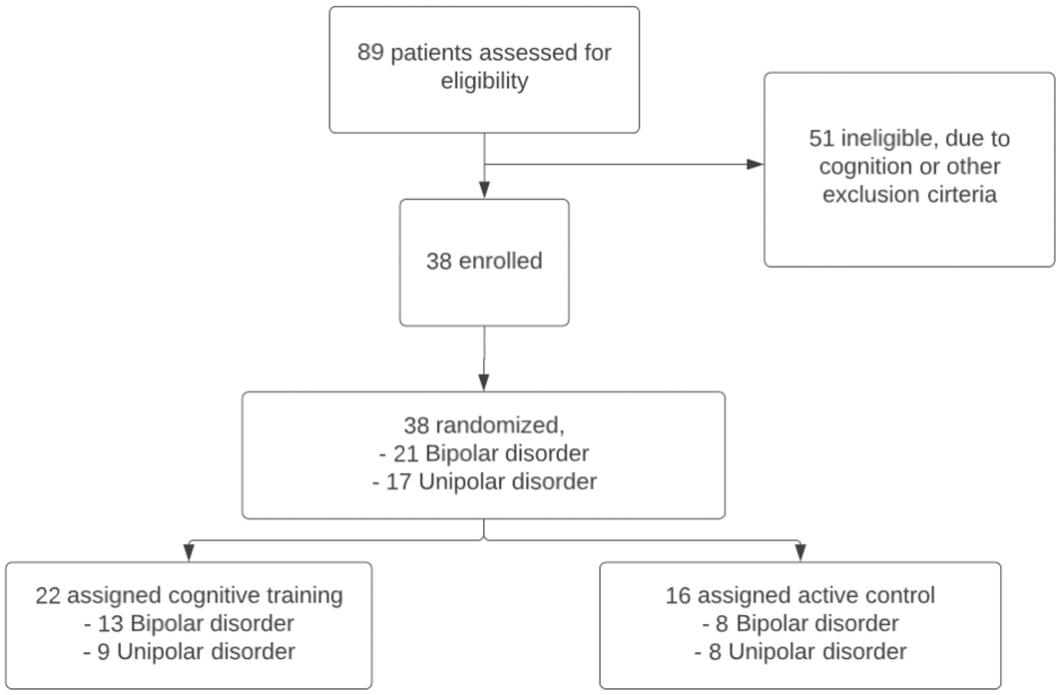
Flow diagram of the enrollment and randomization procedure.

In BrainFit, 89 patients were assessed for eligibility, 38 patients met the inclusion criteria (**Figure 2**). The 38 eligible patients had a mean age of 67.3 years (SD: 5.8), 20 (52.6%) of them were female, and 21 (54.1%) had bipolar disorder. There were no statistically significant differences in demographic and clinical characteristics between the CT and the ACC (**Table 1**). Both groups performed similarly at baseline on global cognitive functioning (MoCA). In both groups patients dropped-out during the study. In the CT group 7 patients and in the ACC group 4 patients did not complete all assessments. We used the intention-to-treat sample for the analyses.

**Table 1:** Demographics and clinical characteristics at baseline of the intention-to-treat sample with late-life mood disorders.

	Intervention N = 22	Active Control N= 16	Group Comparison
**Sex - Female (N (%))**	12 (31.5%)	8 (21.1%)	X^2^(1) = 0.077, p = 0.782
**Age year - Mean(sd)**	66.9 (5.73)	68.0 (6.08)	U = 142.00 p = 0.326
**Diagnosis - (N (%))** - Bipolar - Unipolar	13 (34.2%) 9 (23.7%)	8 (21.1%) 8 (21.1%)	X^2^ (1) = 0.310, p = 0.578
**Current Medication - (N (%))** - SSRI - Tricyclic - Classic MAO inhibitor - Antipsychotic - Lithium - Benzodiazepines - Other mood stabilizers - other	8 (21.1%) 7 (18.4%) 2 (5.3%) 8 (21.1%) 7 (18.4%) 3 (7.9%) 2 (5.3%) 2 (5.3%)	4 (10.5%) 1 (2.6%) 0 5 (13.2%) 5 (13.2%) 3 (7.9%) 6 (15.8%) 5 (13.2%)	p = 0.137
**Education *Verhage* ^a^ *-* (N (%))** - 2 - 4 - 5 - 6 - 7	0 1 (2.6%) 4 (10.5%) 11 (28.9%) 6 (15.8%)	1 (2.6%) 1 (2.6%) 2 (5.3%) 6 (15.8%) 6 (15.8%)	X^2^ (4) = 2.246, p = 0.691
**Ethnicity - (N (%))** - Dutch - Other	- 22 (57.9%) - 0	- 15 (39.5%) - 1 (2.6%)	X^2^ (1) = 1.412, P = 0.235
**Onset depression - (N (%))** - Early *before age of 55* - Late *after age of 55*	- 17 (44.7%) - 5 (13.2%)	- 12 (31.6%) - 4 (10.5%)	X^2^ (1) = 0.026, p = 0.871
**Age first mood episode - Mean (sd)**	36.8 (18.2)	38.3 (18.6)	t(36) = -0.238, p = 0.813
**MoCA - Mean (sd)**	25.1 (2.25)	25.1 (1.85)	t(29) = -0.011, p = 0.991
**CFQ - Mean (sd)**	52.2 (7.16)	53.9 (8.03)	t(35) = -0.698, p = 0.490
**Mansa – Mean (sd)**	52.8 (8.81)	53.4 (8.53)	t(36) = -0.217, p = 0.830
**MADRS - Mean (sd)**	12.8 (8.75)	14.3 (8.16)	t(36) = -0.528, p = 0.600
**Stroop card 1 – Mean (sd)**	39.1 (11.3)	36.33 (8.11)	t(33) = 0.806, p = 0.426
**Stroop card 2 – Mean (sd)**	37.7 (10.4)	35.87 (7.76)	t(33) = 0.573, p = 0.571
**Stroop card 3 – Mean (sd)**	37.8 (6.88)	39.40 (8.05)	t(33) = -0.653, p = 0.518
**Stroop Interference – Mean (sd)**	45.7 (8.82)	49.20 (8.47)	t(33) = -1,181, p = 0.246

MoCA, Montreal Cognitive Assessment, measurement for global cognitive functioning; CFQ, Cognitive Failures Questionnaire, measurement for subjective cognitive functioning; Mansa, Manchester Short Assessment of Quality of life, measurement for quality of life; MADRS, Montgomery Asberg Depression Rating scale, measurement of depression severity.

Scores on the Stroop task are t-transposed scores, corrected for age, sex, and Verhage education level. Group differences in continuous variables were determined by independent samples t-test. If a variable did not meet the assumptions, the non-parametric Mann-Whitney U test was used. Group differences in categorical variables were calculated using chi-squared tests.

^a^ According to Dutch Verhage education classification (range 1: lower than primary school, to 7: university).

### Primary outcome

#### Executive functioning

**Figure 3:**
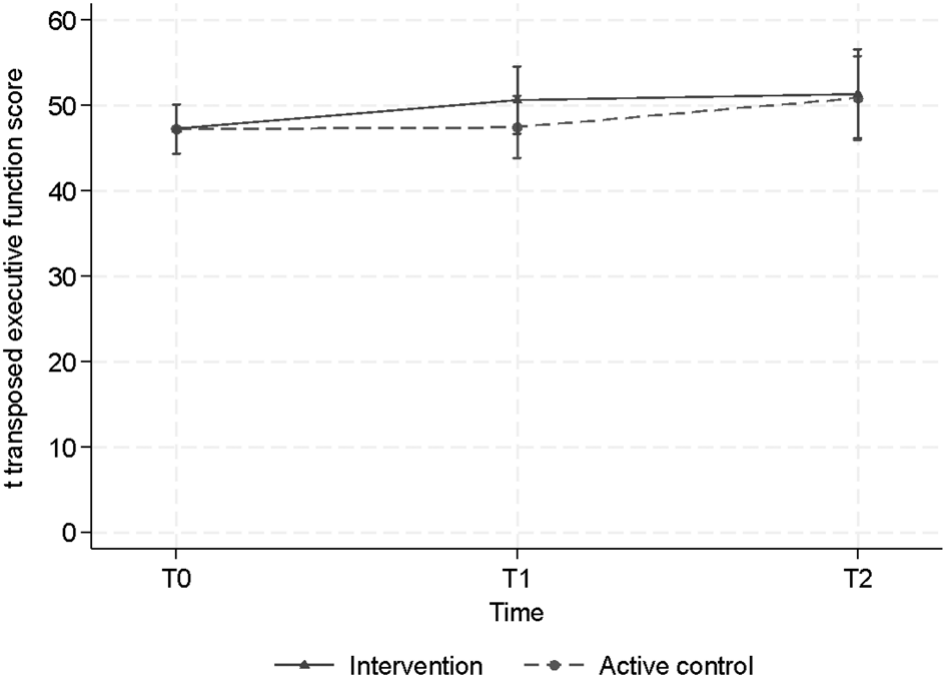
Estimated mean scores, when correcting for baseline differences, of executive functioning across time for the intervention (CT) group and the active control condition (ACC) group.

We did not find statistically significant effects on executive functioning over time in either condition (ACC or CT), nor a statistically significant effect of CT over ACC, as modeled by CT x time interaction, finding only small to moderate effect sizes (**Table 2**). Overall, in both groups, no significant increase in executive functioning was observed over time, and no significant difference was observed between both groups (**Figure 3**).

**Table 2:** Estimated mean levels of “Stroop score” by intervention arm and time of measurement.

	time	Estimated means	s.e.	95%CI	Within group^1)^ test results	Between group^2)^ test results
active control	baseline	47.3	1.5	(44.4, 50.1)		
	post-training	47.5	1.9	(43.8, 51.1)	z=0.1, p=0.904	
	follow-up	50.9	2.5	(46.0, 55.8)	z=1.6, p=0.115	

intervention	baseline	47.3	1.5	(44.4, 50.1)		
	post-training	50.6	2.0	(46.7, 54.6)	z=1.8, p=0.072	z=1.3, p=0.188
	follow-up	51.4	2.7	(46.1, 56.6)	z=1.6, p=0.101	z=0.1, p=0.884

^1)^ test results of comparing measurement at specific time point to measurement at baseline within group.

^2)^ test results of comparing group-by-time interaction effects.

### Secondary outcomes

#### Subjective cognitive complaints

**Figure 4:**
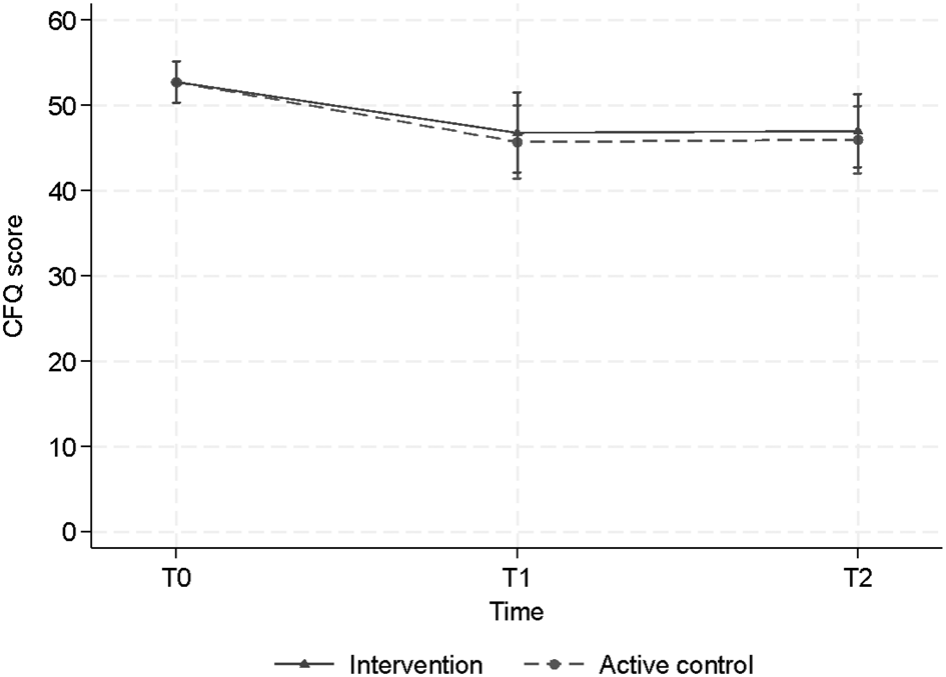
Estimated mean levels of CFQ (with 95% CI’s) by intervention arm and time of measurement.

Overall, a decrease in CFQ score, subjective cognitive failure in daily life, was observed (**Figure 4**). The LMM showed a statistically significant decrease post-training (T1) and at follow-up (T2) in both the CT group and in the ACC group, with effect sizes and p values respectively d=0.8, p=0.003 and 0.8, p= 0.002 (CT) versus d=0.9, p<0.001 and d=0.9, p<0.001 (ACC)). At T1 the CFQ score of the CT group decreased from 52.7 points to 46.8 points and the ACC group decreased from 52.7 to 45.7. At T2 both groups remained stable at 47 and 46 points, respectively. However, neither at T1 nor at T2 we found statistically significant differences between the two groups (T1, p=0.68) and (T2, p = 0.68) (**Table 3**), suggesting that both groups showed less subjective cognitive complaints in daily life over time.

**Table 3:** Estimated mean levels of CFQ by intervention arm and time of measurement.

	time	Estimated means	s.e.	95%CI	Within group^1)^ test results	Between group^2)^ test results
active control	baseline	52.7	1.2	(50.3, 55.1)		
	post-training	45.7	2.2	(41.4, 50.1)	z=-3.9, p<0.001	
	follow-up	46.0	2.0	(42.0, 50.0)	z=-4.0, p<0.001	

intervention	baseline	52.7	1.2	(50.3, 55.1)		
	post-training	46.8	2.4	(42.1, 51.5)	z=-3.0, p=0.003	z=0.4, p=0.682
	follow-up	47.0	2.2	(42.7, 51.3)	z=-3.1, p=0.002	z=0.4, p=0.680

^1)^ test results of comparing measurement at specific time point to measurement at baseline within group.

^2)^ test results of comparing group-by-time interaction effects.

#### Quality of life

**Figure 5:**
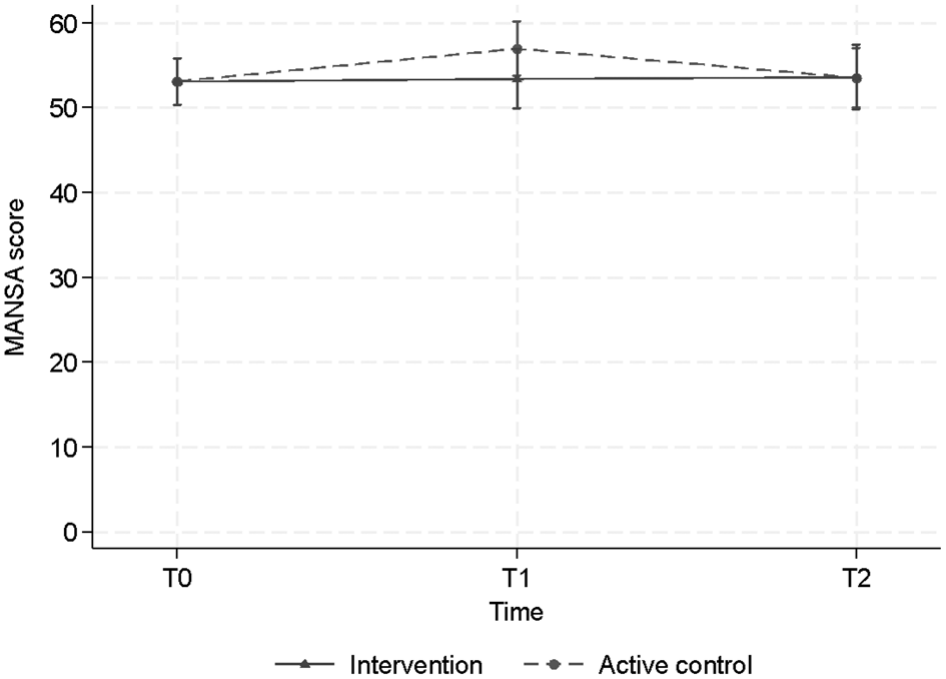
Estimated mean levels of MANSA (with 95% CI’s) by intervention arm and time of measurement.

The Mansa scores, measuring quality of life, showed no significant improvement at T1 in the CT group but did show a significant improvement in the ACC group. At T2 there was no effect of the CT or ACC on the quality of life (**Table 3**; **Figure 5**).

#### Depression severity

The MADRS, measuring depression severity scores, showed low within group effects sizes and correspondingly no statistically significant changes after training. We found no significant effect of time or condition (**Table 3**).

## Discussion

In this study, we assessed the effect of an 8-week online cognitive training (CT), compared to an active control condition (ACC) on executive functioning with a double blind randomized, controlled research design in 38 patients with partly remitted late-life mood disorders. Our results show no beneficial effect of an 8-week computerized CT on the primary outcome; i.e executive functioning. Subjective cognitive functioning did improve in both the CT and the ACC, but no statistically significant difference between-group differences were observed.

Our results are not fully in line with previous studies showing positive effects of CT in various patient groups (16–18, 22, 39). Whereas we found small positive effects of CT on subjective cognitive functioning, we also found these effects in the active control condition. Preiss et al. (12) also found positive effects on executive functioning in both groups, suggesting that the improvement is due to general cognitive activity. Our results partly comply with previous results found by van Balkom et al. (20) using the same online CT in patients with Parkinson’s disease showing a subtle positive effect on mental processing speed; i.e. Participants in the CT group were on average 0.30 SD (i.e., 1.5 s) faster on difficulty load 4 of this task (secondary outcome): 95% CI: -0.55 to -0.06, p = 0.015.

Some differences with previous studies can be due to methodological limitations, such as small sample size, the amount of missing data, or the active control group. Due to COVID-19 a lot of the assessments were performed over the phone, hampering assessment with the STROOP test. This has led to missing data on the primary outcome. By chance, there was more missing data in the CT group than in the ACC group. This could be an explanation for not finding a difference between the groups. Additionally, we cannot exclude that the games in the active control group engage cognitive and executive functioning and thus influence the size of the between-group differences. One possible solution to overcome this would be to add an additional less active condition, such as a waiting list condition or a treatment as usual (TAU) condition. Furthermore, we cannot exclude a learning effect for the STROOP task in our study, despite the gap of 8 weeks and 3 months between the assessments. Another explanation could be the fact that our patients were highly educated (76% of the patients in this study finished higher education) and improvement due to CT may therefore be limited. Finally, our study used the STROOP task in solitude to measure executive functioning. Despite the STROOP task measuring multiple aspects of executive function, it would be good to validate the results with tasks measuring other aspects of executive functioning as it refers to an umbrella term of mental skills to manage oneself in order to achieve goals. A possibility is using a composite score of different tasks measuring executive functions, [e.g., the STROOP (30), the Trail Making Test form B (40), and the Tower of London (41)].

### Strengths and weaknesses

The results of this study should be seen in the light of its strengths and limitations. The current study was performed using an optimal RCT design. The sample size was calculated based on a large effect size of a previous study, this may have led to a sample size too small to detect significant differences. Patients trained three times a week for eight weeks. Maybe this period was too short to result in a significant improvement of executive functioning. Also, COVID-19 interfered with the study, leading to incomplete data and online assessments instead of face-to-face assessments at the outpatient clinic.

## Conclusion

In conclusion, the online cognitive training, compared to an active control condition, did not improve executive functioning as we hypothesized. Further research is necessary to investigate if CT can be useful in clinical settings. The effects on subjective cognitive functioning shown by both, online CT and the ACC, are persistent and present 3 months after ending the training. Since patients experience improvements in everyday functioning, introducing CT to patients with LLM does seem favorable, possibly combined with additional training (e.g., strategy training, aerobe physical training, or skill training) (42, 43).

## Data Availability

The raw data supporting the conclusions of this article will be made available by the authors, without undue reservation.
